# Barriers to and facilitators of point-of-care ultrasound utilization among physicians, nurse practitioners, and nurses in Japan: a comparative study

**DOI:** 10.1186/s13089-025-00399-4

**Published:** 2025-01-10

**Authors:** Toru Yamada, Takuma Kimura, Kyoko Shigetomi, Takahiro Shinohara, Shuji Ouchi, Suguru Mabuchi, Tomoko Kusama, Takeshi Ishida, Masayoshi Hashimoto

**Affiliations:** 1 Department of General Medicine, Graduate School of Medical and Dental Sciences, Institute of Science Tokyo, 1-5-45 Yushima, Bunkyo-ku, Tokyo 113-8510 Japan; 2 Department of R&D Innovation for Home Care Medicine, Graduate School of Medical and Dental Sciences, Institute of Science Tokyo, Bunkyo-ku, Tokyo 113-8510 Japan; 3 Department of Cardiovascular Surgery, Juntendo University Hospital, Bunkyo-ku, Tokyo 113-0033 Japan; 4 Oita University of Nursing and Health Sciences, Oita city, Oita 870-1201 Japan; 5 Department of Community Medicine (Ibaraki), Graduate School of Medical and Dental Sciences, Institute of Science Tokyo, Bunkyo-ku, Tokyo 113-8510 Japan

**Keywords:** Point-of-care ultrasound, Facilitators, Barriers, Nurse practitioner, Nurse

## Abstract

**Background:**

Point-of-care ultrasound (POCUS) is a valuable skill for generalist physicians, nurse practitioners (NPs), and nurses; however, its utilization remains limited. This study was performed to investigate the current status, barriers, and facilitators of POCUS implementation among physicians, NPs, and nurses in family and hospital medicine in Japan and to identify differences in influencing factors between physicians and NPs/nurses.

**Results:**

A web-based survey was distributed via the mailing lists of four major academic societies in general medicine in Japan—the Japanese Society of Hospital General Medicine, the Japan Primary Care Association, the Japanese Association for Home Care Medicine, and the Japan Society of Nurse Practitioner—from April to June 2024. The respondents included physicians, NPs, and nurses affiliated with these societies. Responses from other professions, duplicate entries, and incomplete surveys were excluded from the analysis, resulting in 913 valid responses (692 physicians and 221 NPs/nurses). Physicians reported a higher POCUS implementation rate than NPs/nurses (73.0 vs. 63.4%, *p* = 0.006). The top two barriers were consistent across both groups: lack of training opportunities (*p* = 0.385) and lack of image acquisition skills (*p* = 0.369). However, NPs/nurses reported significantly greater barriers than did physicians, including lack of mentors (*p* < 0.001), lack of interpretation skills (*p* = 0.007), lack of confidence (*p* < 0.001), poor access to ultrasound devices (*p* < 0.001), and absence of institutional guidelines (*p* < 0.001). The top facilitators for both groups were good access to ultrasound devices (*p* = 0.078) and increased training opportunities (*p* = 0.240), with no significant differences between them. Compared with physicians, NPs/nurses expressed a significantly higher demand for nearby mentors (*p* < 0.001), institutional support (*p* < 0.001), and POCUS certification (*p* = 0.005).

**Conclusions:**

There is currently a lack of POCUS training opportunities across all professional roles. To promote POCUS adoption among NPs and nurses, it is essential to develop mentorship programs, establish institutional guidelines, and create an environment that enables NPs and nurses to perform POCUS confidently through measures such as certification programs.

**Supplementary Information:**

The online version contains supplementary material available at 10.1186/s13089-025-00399-4.

## Background

Point-of-care ultrasound (POCUS) is a focused ultrasound examination designed to assess specific clinical concerns using simplified diagnostic criteria. This approach allows for a shorter learning curve while maintaining diagnostic quality. However, as with other diagnostic tools, it is also essential to note that without adequate training and a correct understanding of its limitations, errors may occur. [1]. POCUS emerged around 2010, initially gaining traction among emergency physicians and intensivists. In recent years, it has increasingly been incorporated into the training of internists, nurse practitioners (NPs), nurses, and medical students [1, 2]. Advances in technology have led to smaller, more advanced, and affordable ultrasound devices, enabling the use of portable machines across various settings. Consistent with its original purpose, POCUS has the potential to become a valuable skill for family physicians and hospitalists working in primary care. However, despite its utility for generalists, POCUS adoption among hospitalists and family physicians is not as widespread as it is among emergency physicians and intensivists [3–8]. Reported barriers to POCUS implementation include limited training opportunities, a lack of nearby mentors, restricted access to ultrasound devices, and variability in skill levels among practitioners [4–8].

Given its characteristics, POCUS is particularly useful for patient assessment by NPs and nurses. Studies involving NPs and advanced practice registered nurses in emergency and critical care settings have shown that POCUS can improve the accuracy of clinical reasoning and enhance the effectiveness of early interventions [9–12]. The importance of accurate clinical judgment using POCUS has been emphasized for NPs and nurses who frequently provide first-contact care [13, 14]. To promote the broader use of POCUS, it is crucial to extend its application to a wider range of healthcare professionals, including general practice physicians, NPs, and nurses. This expansion requires identifying barriers to POCUS mastery and understanding the factors that facilitate its dissemination.

This study was performed to investigate the current status of POCUS utilization; the barriers and facilitators of POCUS among physicians, NPs, and nurses involved in family and hospital medicine in Japan; and the differences in influencing factors between physicians and NPs/nurses.

## Methods

### Participants and setting

Nineteen core medical specialties have been established in Japan. Among these specialties is general practice, which comprises two subspecialties: family medicine and hospital medicine. This study targeted healthcare professionals (including NPs and nurses) working in the field of general practice. A web-based survey on POCUS utilization, barriers, and facilitators was distributed from April to June 2024 via the mailing lists of four major academic societies in general practice: the Japanese Society of Hospital General Medicine, the Japan Primary Care Association, the Japanese Association for Home Care Medicine, and the Japan Society of Nurse Practitioner. Most members of the Japanese Society of Hospital General Medicine, the Japan Primary Care Association, and the Japanese Association for Home Care Medicine are physicians, but NPs and nurses are also included as members.

In Japan, NP certification is overseen by the Japanese Organization of Nurse Practitioner Faculties. To qualify as an NP, a nurse must have at least 5 years of clinical experience after obtaining their nurse license, complete more than 55 credits of graduate-level education in a master’s program, and pass the certification examination administered by the Japanese Organization of Nurse Practitioner Faculties [15, 16]. Separately, since 2015, a training system for nurses for specific medical acts has been stipulated in “Japanese Nursing ACT”. It allows nurses who have received training at designated training institutions to perform specific medical practices such as arterial blood sampling and the insertion of peripherally inserted central catheters under the comprehensive instructions by the physician [15, 17]. This training system for nurses for specific medical acts does not require a master’s degree. NPs, nurses who have completed the training program for specific medical acts, and general nurses are all permitted to perform ultrasound examinations, but they must do so under physician supervision.

This study included physicians, NPs, and nurses (regardless of whether they had completed the training program for specific medical acts) who were members of the four aforementioned academic societies, received survey invitations via the mailing lists, and provided consent to participate. Individuals from other professions, those who responded multiple times, and those who did not complete all survey questions were excluded.

### Survey questionnaire

A web-based survey was distributed via email to collect information on the participants’ backgrounds, including age, sex, profession, years since graduation, affiliated institution and department, and usual scope of practice. Respondents were also asked about their use of POCUS in daily practice, the specific POCUS applications they believed were important to acquire, and the barriers and facilitators they encountered when performing POCUS. Multiple choice selection was allowed for questions on POCUS applications, barriers, and facilitators. A list of the survey questions used for analysis in this study is provided in Supplementary File 1.

### Statistical analysis

Comparisons of participant characteristics, barriers, and facilitators between physicians and NPs/nurses were conducted using the Wilcoxon rank-sum test for continuous variables and the chi-square test for categorical variables. Statistical significance was defined as a *p*-value of <0.05. All data were analyzed using STATA software version 17.0 (StataCorp LLC, College Station, TX, USA).

## Results

### Participants

Responses were obtained from 946 participants, of whom 33 were excluded for not meeting the inclusion criteria (duplicate responses in 24, lack of consent to participate in 5, data entry errors in 3, and non-physician/NP/nurse in 1). The analysis included 913 respondents: 692 physicians, 163 NPs, and 58 nurses (14 of whom had completed training program for specific medical acts). The average number of years since graduation was 22.6 (standard deviation, 10.9). Approximately 43.1% of respondents worked in clinics and 42.4% worked in community hospitals, with each group representing around 40% of the total. The most common primary specialty was family medicine, followed by hospital medicine. Overall, 80% of respondents were involved in outpatient care, 45% in inpatient care, and 19.5% in critical care (Table 1).

**Table 1:** Participants’ characteristics

Category	Total (n = 913)	Physicians (n = 692)	NPs/nurses (n = 221)
Age, years	47.7 ± 10.6	48.7 ± 11.0	44.7 ± 8.3
Male	620 (67.9)	547 (79.1)	73 (33.0)
Postgraduate years	22.6 ± 10.9	22.2 ± 11.4	21.2 ± 9.1
Institution			
Clinic/home care station	394 (43.1)	339 (49.0)	55 (24.9)
Community hospital	387 (42.4)	264 (38.2)	123 (55.7)
University hospital	123 (13.5)	86 (12.4)	37 (16.7)
Others	9 (1.0)	3 (0.4)	6 (2.7)
Department			
Hospital medicine	260 (28.5)	225 (32.5)	35 (15.8)
Family medicine	369 (40.4)	318 (46.0)	51 (23.1)
IM subspecialties	107 (11.7)	76 (11.0)	31 (14.0)
EM, CCM	61 (6.7)	17 (2.5)	44 (19.9)
Surgical specialties	86 (9.4)	42 (6.1)	44 (19.9)
Others	30 (3.3)	14 (2.0)	16 (7.2)
Clinical field*			
Inpatient care	413 (45.2)	285 (41.2)	128 (57.9)
Critical care	178 (19.5)	96 (13.9)	82 (37.1)
Outpatient care	731 (80.1)	620 (89.6)	111 (50.2)
Home visit care	535 (58.6)	461 (66.6)	74 (33.5)

Data are presented as n (%) or mean ± standard deviation

*NP* nurse practitioner, *Department* primary department of physicians, nurses, and nurse practitioners, *IM* internal medicine, *EM* emergency medicine, *CCM* critical care medicine

*Multiple choices allowed

### POCUS use in daily clinical practice

**Fig. 1:**
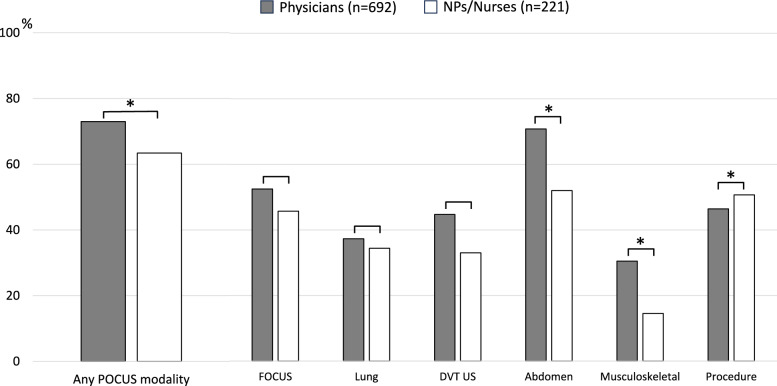
POCUS use in daily clinical practice. *NP* nurse practitioner, *FOCUS* focused cardiac ultrasound, *POCUS* point-of-care ultrasound, *DVT US* deep vein thrombosis ultrasound. *p < 0.05

Figure 1 shows the implementation status and types of POCUS performed in daily clinical practice. A total of 505 (73.0%) physicians and 140 (63.4%) NPs/nurses used some form of POCUS in their routine clinical practice, with a significantly higher proportion among physicians than among NPs/nurses (73.0 vs. 63.4%, *p* = 0.006). The proportions of physicians and NPs/nurses performing focused cardiac ultrasound (FOCUS), lung ultrasound, and deep vein thrombosis (DVT) ultrasound were 52.5, 37.3, and 44.7% for physicians and 45.7, 34.4, and 33.0% for NPs/nurses, respectively, with no significant differences between the groups. However, for abdominal and musculoskeletal ultrasound, physicians had a higher proportion of users (70.8 vs. 52.0% and 30.5 vs. 14.5%, respectively; both *p* < 0.001). Conversely, the proportion of users was higher among NPs/nurses for ultrasound-guided procedures (50.7 vs. 46.4%, *p* < 0.001).

**Fig. 2:**
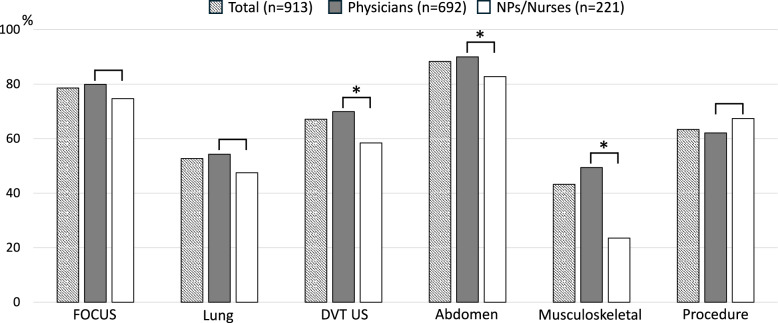
POCUS items that should be mastered in daily clinical practice. *NP* nurse practitioner, *FOCUS* focused cardiac ultrasound, *DVT US* deep vein thrombosis ultrasound. *p < 0.05

Figure 2 illustrates the POCUS applications that participants considered necessary for their routine work environment. The top two applications were the same for both physicians and NPs/nurses: abdominal ultrasound and FOCUS. However, the third most frequently cited application differed between the groups, with DVT ultrasound ranking third for physicians and procedures for NPs/nurses. There were no statistically significant differences between physicians and NPs/nurses regarding the need to acquire FOCUS, lung, and procedural skills (79.9 vs. 74.7%, 54.3 vs. 47.5%, and 62.1 vs. 67.4%, respectively). By contrast, significantly more physicians than NPs/nurses reported the need to acquire DVT, abdominal, and musculoskeletal ultrasound skills (69.9 vs. 58.4%, *p* = 0.001; 90.0 vs. 82.8%, *p* = 0.004; and 49.4 vs. 23.5%, *p* < 0.001, respectively).

### Barriers to POCUS use

**Fig. 3:**
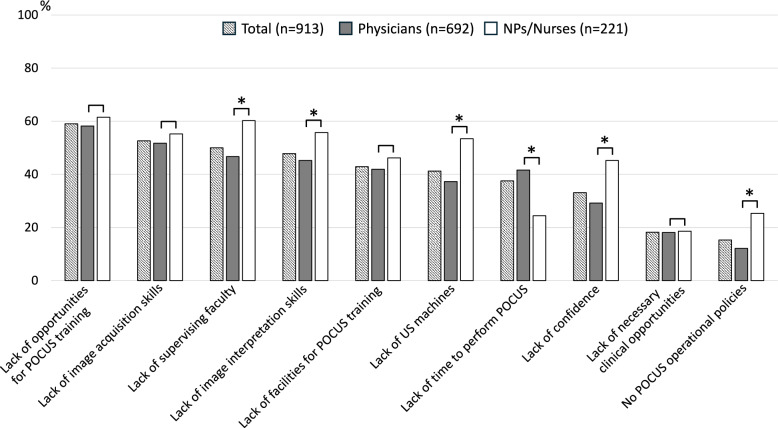
Top 10 barriers to the use of POCUS: a comparison between physicians and NPs/nurses. *NP* nurse practitioner, *POCUS* point-of-care ultrasound, *US* ultrasound. *p < 0.05

Figure 3 shows the top 10 barriers to POCUS implementation. There were no significant differences between physicians and NPs/nurses for the top two barriers: lack of POCUS training opportunities (*p* = 0.385) and insufficient image acquisition skills (*p* = 0.369). However, NPs/nurses were significantly more likely than physicians to report a lack of mentors (3rd, *p* < 0.001), insufficient image interpretation skills (4th, *p* = 0.007), and lack of confidence (8th, *p* < 0.001) as barriers.

Regarding environmental factors, NPs/nurses were also significantly more likely to cite poor access to ultrasound devices (6th, *p* < 0.001) and the absence of established POCUS protocols at their facilities (10th, *p* < 0.001) as barriers. Although the perceived need for POCUS in clinical practice was similar between the groups (9th, *p* = 0.870), lack of time was reported as a significantly greater barrier for physicians (7th, *p* < 0.001).

### Facilitators of POCUS use

**Fig. 4:**
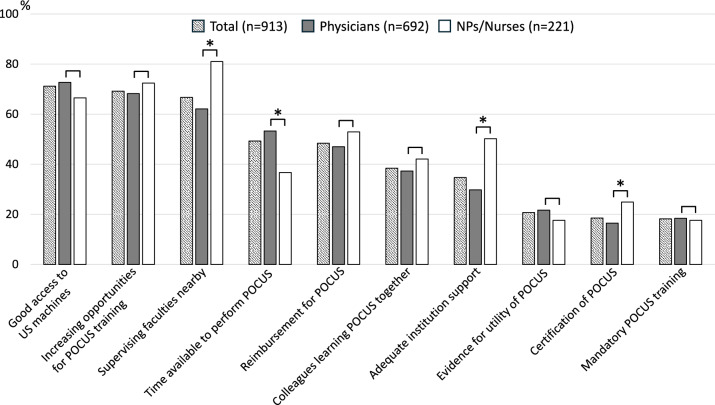
Top 10 facilitators to the use of POCUS: a comparison between physicians and NPs/nurses. *NP* nurse practitioner, *POCUS* point-of-care ultrasound, *US* ultrasound. *p < 0.05

Figure 4 presents the top 10 facilitators for POCUS implementation. Both physicians and NPs/nurses identified good access to ultrasound devices (1st, *p* = 0.078) and increased POCUS training opportunities (2nd, *p* = 0.240) as the top two facilitators, with no significant differences between the groups. However, NPs/nurses were significantly more likely than physicians to express a need for nearby mentors (3rd, *p* < 0.001), support from their affiliated institutions (7th, *p* < 0.001), and POCUS certification (9th, *p* = 0.005). Approximately half of the respondents cited reimbursement for POCUS procedures as a facilitator.

## Discussion

This study investigated the barriers and facilitators to POCUS implementation among 913 physicians, NPs, and nurses. POCUS was used in daily clinical practice by 73.0% of physicians and 63.4% of NPs/nurses. There were no significant differences between physicians and NPs/nurses in the proportions performing FOCUS, lung, or DVT ultrasound. However, the proportion was significantly higher for physicians in abdominal and musculoskeletal ultrasound, while NPs/nurses had a higher proportion for ultrasound-guided procedures. The rankings of essential POCUS skills were similar for both groups, with lung (5th) and musculoskeletal (6th) ultrasound rated as lower priorities among the six skills.

Compared with physicians, barriers for NPs/nurses were significantly more closely associated with a lack of mentors, insufficient image interpretation skills, lack of confidence, limited access to equipment, and the absence of clear policies. In terms of facilitators, NPs/nurses expressed a greater need for support from nearby mentors, adequate institutional support, and official certification of POCUS skills than did physicians. These findings suggest that environmental factors, such as equipment availability, institutional policies, and mentorship, have a greater influence on NPs and nurses than on physicians when implementing POCUS.

### Study population and frequency of POCUS use

In this survey, 70.7% of all respondents (physicians: 73.0%, NPs/nurses: 63.4%) reported using some form of POCUS in their routine clinical practice. Among physicians, the most commonly performed POCUS was abdominal ultrasound (70.8%), followed by FOCUS (52.5%), procedures (46.4%), and DVT ultrasound (44.7%). Utilization rates for lung ultrasound (37.3%) and musculoskeletal ultrasound (30.5%) were both in the 30% range. These rates are higher than those in previous reports in the fields of primary care and hospital medicine, which were the main areas of practice for the respondents, as well as past reports from Japan [4, 5, 7, 8]. Based on these findings, it is considered that this survey reflects the perspectives of a group of physicians who actively perform POCUS.

Few studies to date have addressed POCUS implementation by NPs or nurses. POCUS is commonly used by NPs and advanced practice registered nurses in emergency and critical care settings for confirming endotracheal intubation, performing pediatric lung ultrasound, and evaluating ectopic pregnancies [9, 10, 12, 18]. It is also used at the bedside for guiding hemodialysis access and evaluating the inferior vena cava [11, 19]. The need for POCUS is increasingly being emphasized in the care of older adults, a field that is expected to grow rapidly in aging societies [13]. However, the extent to which POCUS is used in each of these areas remains unclear, making it difficult to compare the frequency of POCUS use by NPs and nurses in this survey with that in previous reports. Nonetheless, the fact that the reported frequency in this study does not significantly differ from that of physicians suggests that the responses came from a group of NPs and nurses who actively perform POCUS.

The most common primary specialty for both physicians and NPs/nurses was family medicine, with 80% of respondents working in clinics or community hospitals and more than half engaged in outpatient care. These findings suggest that the responses reflect a group of physicians and NPs/nurses who actively use POCUS in primary care settings.

Both the implementation frequency and perceived necessity for lung and musculoskeletal ultrasound were low. In a survey of 241 participants from 62 low- and middle-income countries (excluding Japan), lung ultrasound was the most frequently indicated modality (58%), followed by cardiac, abdominal, and vascular ultrasound [20]. In the field of hospital medicine in the United States, lung ultrasound is used at a frequency comparable to that of abdominal ultrasound [7]. However, a previous report from Japan indicated that the frequency of lung ultrasound was 5% before training and 17% after training, which was lower than the frequency of other modalities [8]. In this study, the frequency of lung ultrasound was in the 30% range but still lower than that of other modalities. This relatively lower frequency of lung ultrasound in Japan may be a unique characteristic, and the reasons for this are unclear, warranting further investigation.

The proportion of users was higher among NPs/nurses for ultrasound-guided procedures (50.7 vs. 46.4%, *p* < 0.001). Several reasons may explain this. First, among respondents, the largest proportion of physicians (46.0%) worked in family medicine departments (compared to 23.1% for NPs/nurses), while NPs/nurses had a combined total of 39.8% in EM, CCM, and surgical specialties (compared to only 8.6% for physicians). This suggests that NPs/nurses may have had more opportunities to perform procedures. Additionally, PICC placement is considered a key procedure for NPs [15, 17], which may lead them to take a more active role as practitioners in settings where NPs are present. However, further investigation is needed to confirm this.

### Barriers and facilitators

The most frequently cited barrier was the lack of POCUS training opportunities, consistent with previous reports from multiple countries indicating that this is a major factor hindering POCUS utilization [6–8, 20, 21]. No significant differences were observed between physicians and NPs/nurses. Factors related to acquiring POCUS competency include image acquisition skills, image interpretation skills, and confidence in performing the procedure [22, 23]. In this survey, a lack of image acquisition skills was the second most common barrier, with no significant differences between physicians and NPs/nurses. However, image interpretation skills and confidence were significantly greater barriers for NPs/nurses than for physicians.

POCUS is most effective when images are interpreted alongside other clinical findings and the patient’s medical history [1]. Therefore, differences between physicians and NPs/nurses in skills and confidence in image interpretation may stem from variations in training backgrounds related to history-taking and pathophysiology, as well as differences in focus and priorities during routine clinical practice. [9, 18]. Additionally, because NPs/nurses may perform ultrasound less frequently than physicians, their lack of familiarity with conducting POCUS could contribute to anxiety about performing the procedure. Previous studies have shown that a lack of confidence is a barrier to POCUS implementation among nurses [19]. Furthermore, in this survey, a lack of supervisors and unclear institutional policies were found to be significantly greater barriers for NPs/nurses than for physicians, emphasizing the importance of having accessible supervisors and institutional or certification program support as facilitators. It is presumed that many institutions lack established protocols for NPs and nurses to perform POCUS independently [12], and these environmental factors may further contribute to their lack of confidence.

Acquiring POCUS skills can enable NPs and nurses to perform earlier and more accurate screening [9–11]. Additionally, studies have shown that when NPs and nurses participate in the same training courses as physicians, they can achieve similarly effective educational outcomes [12, 14]. Establishing new, separate courses specifically for NPs and nurses could be costly and may be unnecessary; instead, making existing physician-targeted courses more accessible to them is recommended. Given the clinical utility of POCUS training for NPs and nurses, the implementation of formal education programs for these practitioners is warranted [9–11, 13, 18].

Access to high-quality ultrasound machines was identified as the top facilitator, while NPs and nurses perceived the lack of such machines as a significantly greater barrier than did physicians. Limited access to ultrasound machines is a major issue not only in Japan but also in many other countries [4, 5, 7, 8, 20]. This problem is less pronounced in settings such as the intensive care unit, where ultrasound machines are readily available, but it remains a significant barrier in general wards and outpatient clinics [3]. Even for physicians, access to ultrasound machines can be challenging; thus, it is unsurprising that this issue poses an even greater barrier for NPs/nurses. Previous studies have also highlighted this concern [11].

## Limitations

This study investigated the barriers and facilitators to POCUS implementation among physicians and NPs/nurses; the results do not reflect the overall prevalence of POCUS usage in Japan. Because the data were collected through an anonymous, voluntary email survey, there is a potential bias toward respondents with a strong interest in POCUS. Additionally, approximately 75% of the nurse respondents were NPs, so the results may not represent the trends among the entire nursing workforce. Approximately 70% of the respondents, both physicians and NPs/nurses, were already using POCUS. While this provides valuable insights into the barriers and facilitators for broader POCUS adoption, it may limit the generalizability of the findings to those who have not yet adopted POCUS.

## Conclusion

The results of this study suggest that while physicians and NPs/nurses share certain common barriers and facilitators to POCUS implementation, environmental factors may have a more significant impact on NPs/nurses. In addition to the general lack of POCUS training opportunities and ultrasound equipment across professions, NPs/nurses face specific challenges, such as a shortage of mentors due to the small number of practitioners, lack of confidence, and unclear institutional policies regarding POCUS use. Given the immediacy, non-invasiveness, ease of learning, and reliability of ultrasound examinations, POCUS is inherently well-suited for non-physician healthcare professionals. In the context of team-based medicine that emphasizes interdisciplinary collaboration, equipping NPs and nurses with POCUS skills could be an effective strategy to expand their scope of practice and maintain the quality of care. While adequate training and supervision are essential, it is also crucial to create an environment where NPs and nurses can confidently perform POCUS by developing mentorship programs, establishing clear institutional policies, and implementing certification systems.

## Supplementary Information

Additional file 1.

## Data Availability

The datasets used and/or analyzed during the current study are available from the corresponding author upon reasonable request.

## Abbreviations

POCUS: Point-of-care ultrasound
NP: Nurse practitioner
FOCUS: Focused cardiac ultrasound
DVT: Deep vein thrombosis
