# Supplementary material for: Barriers to and facilitators of point-of-care ultrasound utilization among physicians, nurse practitioners, and nurses in Japan: a comparative study
Source: Ultrasound J. 2025 Jan 10;17:1. doi: 10.1186/s13089-025-00399-4 (PMC11723860; doi:10.1186/s13089-025-00399-4)
Supplement: Supplementary file 1 — Additional file 1. [file 13089_2025_399_MOESM1_ESM.docx]

**Supplementary File 1:** Survey Questionnaire

| **Questions** |  |
| --- | --- |
| **Background** |  |
| 1. Are you a physician, nurse, or nurse practitioner? |  |
| 2. Is this your first time answering this survey? |  |
| 3. What is your age? |  |
| 4. What is your gender? |  |
| 5. What is your occupation? |  |
| 6. What year did you graduate from medical school or nursing school? |  |
| 7. What is the main type of facility where you work? |  |
| 8. What is the main department you are affiliated with at your workplace? |  |
| 9. Do you regularly provide medical care or nursing care to inpatients in general wards? |  |
| 10. Do you regularly provide medical care or nursing care to inpatients in intensive care settings? |  |
| 11. Do you regularly provide medical care or nursing care to outpatients? |  |
| 12. Do you regularly provide home care or nursing? |  |

| **POCUS Use in Daily Clinical Practice** |  |
| --- | --- |
| 13. Do you perform any kind of POCUS in your daily clinical practice? |  |
| 14. Do you perform FOCUS in your daily clinical practice? |  |
| 15. Do you perform lung ultrasound in your daily clinical practice? |  |
| 16. Do you perform DVT ultrasound in your daily clinical practice? |  |
| 17. Do you perform abdominal ultrasound in your daily clinical practice? |  |
| 18. Do you perform musculoskeletal ultrasound in your daily clinical practice? |  |
| 19. Do you perform procedure ultrasound in your daily clinical practice? |  |
| 20. Which POCUS applications do you believe should be mastered in daily clinical practice? |  |

| **Barriers and Facilitators to the Use of POCUS** |  |
| --- | --- |
| 21. What are the barriers to performing POCUS in daily practice? |  |
| 22. What are the facilitators to performing POCUS in daily practice? |  |

POCUS, point-of-care ultrasound; FOCUS, focused cardiac ultrasound; DVT US, deep vein thrombosis ultrasound
